# Plastidial phosphoglucose isomerase undergoes thioredoxin-mediated redox modification without altering catalytic activity

**DOI:** 10.1042/BCJ20253425

**Published:** 2025-12-17

**Authors:** Subaru Nishide, Kosuke Fujii, Keisuke Yoshida

**Affiliations:** 1 Laboratory for Chemistry and Life Science, Institute of Integrated Research, Institute of Science Tokyo, Yokohama, Japan; 2 School of Life Science and Technology, Institute of Science Tokyo, Yokohama, Japan

**Keywords:** *Arabidopsis thaliana*, phosphoglucose isomerase, redox modification, thioredoxin

## Abstract

Thioredoxin (Trx)-mediated redox regulation is a posttranslational mechanism that controls enzyme activity by reversibly switching the oxidation/reduction states of Cys residues. In plant cells, numerous enzymes across diverse biological systems have been suggested as targets of redox regulation; however, a complete understanding is lacking. In this study, we report that phosphoglucose isomerase (PGI) in plastids represents a novel class of redox-sensitive enzymes. PGI catalyzes the reversible interconversion of fructose 6-phosphate and glucose 6-phosphate and operates at the branch point between the Calvin–Benson cycle and the starch synthesis pathway in plastids. Using an affinity chromatography-based method, we found that plastidial PGI physically interacts with Trx in a redox-dependent manner. *In vitro* assays with recombinant proteins from *Arabidopsis thaliana* revealed that plastidial, but not cytosolic, PGI forms an intramolecular disulfide bond. Among plastid-localized Trx subtypes, the *f*- and *m*-types were more effective in reductively cleaving the disulfide bond. MS-based peptide mapping, site-directed mutagenesis, and structural modeling identified the redox-active Cys pair. Furthermore, *in vivo* analysis using *Arabidopsis* leaves showed that plastidial PGI is converted from oxidized to reduced states upon illumination, which absolutely depends on the Trx system. Notably, despite these redox modifications, PGI catalytic activity remained nearly identical in both states. Although PGI activity was affected by some metabolites and pH, it showed no sensitivity to redox state. Our findings demonstrate that plastidial PGI is a redox-sensitive enzyme but functionally uncoupled from activity modulation.

## Introduction

Thiol-based redox regulation is a posttranslational protein modification that controls enzyme activity by switching the oxidation/reduction states of Cys residues (e.g., disulfide bond formation/cleavage). A small, ubiquitous protein thioredoxin (Trx) is primarily responsible for this regulation. Trx contains a highly conserved WCGPC amino acid sequence at its active site. Using the two Cys residues in this sequence, Trx catalyzes a dithiol–disulfide exchange reactions with target enzymes, modulating their catalytic activities. Thus, Trx is essential for transmitting reducing power to redox-sensitive enzymes and adjusting cellular functions in response to local redox changes [1,2].

 Although Trx-mediated redox regulation occurs in all organisms, including prokaryotes and eukaryotes, its function in plant chloroplasts is uniquely linked to light. Upon illumination, the thylakoid membrane converts light energy into reducing power *via* photosynthetic electron transport. Trx acquires this reducing power from photosynthetically reduced ferredoxin (Fd) through Fd-Trx reductase (FTR) and transfers it to redox-sensitive Trx-targeted enzymes. Typically, these enzymes switch from inactive to active forms when specific disulfide bonds are reductively cleaved. This Fd/Trx pathway enables light-responsive activation of chloroplast enzymes and has been regarded as the core mechanism of redox regulation in chloroplasts [3,4].

 Another characteristic of chloroplast redox regulation is the presence of multiple Trx subtypes: *f*-, *m*-, *x*-, *y*-, and *z*-types [5,6]. These subtypes differ in their redox potentials and protein surface charges, potentially conferring functional diversity, such as distinct target recognition mechanisms [7–10]. Additional regulatory components have been identified in chloroplasts. The most well-known is NADPH-Trx reductase C (NTRC), which contains both an NADPH-Trx reductase domain and a Trx domain within a single polypeptide [11]. NTRC uses NADPH as a reducing source, functioning independently from the canonical Fd/Trx pathway. These findings support the emerging hypothesis that chloroplasts possess a complex redox network sustained by divergent pathways of reducing power, allowing flexible and sophisticated control of chloroplast functions. Nonetheless, understanding this network remains a major challenge in plant redox regulation research [12,13].

 A key challenge is achieving a consistent understanding of which chloroplast enzymes are redox-regulated. Classically, only a limited number of chloroplast enzymes (e.g., four enzymes in the Calvin–Benson cycle) were recognized as targets of redox regulation [3,4]. However, advances in proteomics-based strategies have helped reveal many more enzymes as potential targets. For example, an affinity chromatography-based method using a monocysteine mutant of Trx as bait has enabled systematic screening for Trx-interacting enzymes [14]. In recent years, novel Cys-labeling techniques have identified a wide array of chloroplast enzymes as redox-sensitive to environmental stimuli [15–17]. Undoubtedly, these redox-proteomics approaches have offered valuable insights into target enzymes of redox regulation. However, our consistent understanding remains limited because large-scale proteomics-based strategies cannot assess how enzyme activity is affected by redox state changes. Furthermore, most of the proteomics approaches do not evaluate key aspects of redox regulation, such as Trx subtype specificity and redundancy. To overcome these limitations, redox regulation must be examined for individual enzymes using biochemical methods.

 Phosphoglucose isomerase (PGI) catalyzes the reversible interconversion of fructose 6-phosphate (F6P) and glucose 6-phosphate (G6P). Typically, plants possess two types of PGI, one localized to plastids and the other to the cytosol [18,19]. In plastids, PGI functions in the initial step of the starch synthesis pathway, branching off from the Calvin–Benson cycle. Accordingly, an *Arabidopsis* mutant with a decreased plastidial PGI activity exhibited a deficiency in leaf starch synthesis [18]. Plastidial PGI was also shown to be key to the production of precursors for isoprenoid-derived hormones and storage reserves [20,21]. Given these important roles in carbon metabolism, it is reasonable to postulate that plastidial PGI should be tightly regulated. Recent redox-proteomics studies have identified plastidial PGI as redox-sensitive, with its thiol status altered in response to light conditions [16,17]. These findings imply that plastidial PGI is subject to redox regulation, although definitive conclusions are lacking.

 In this study, we investigated the potential redox regulation of plastidial PGI primarily through biochemical approaches. We found that (i) the enzyme dynamically modifies its redox state in a Trx-dependent manner both *in vitro* and *in vivo*; (ii) a specific Cys pair is essential for dithiol/disulfide conversion; and (iii) redox modification does not lead to any changes in catalytic activity. These results identify plastidial PGI as a novel enzyme subject to Trx-mediated redox modification without catalytic activity change.

## Results

### Plastidial PGI interacts with Trx in a redox-dependent manner

**Figure 1:**
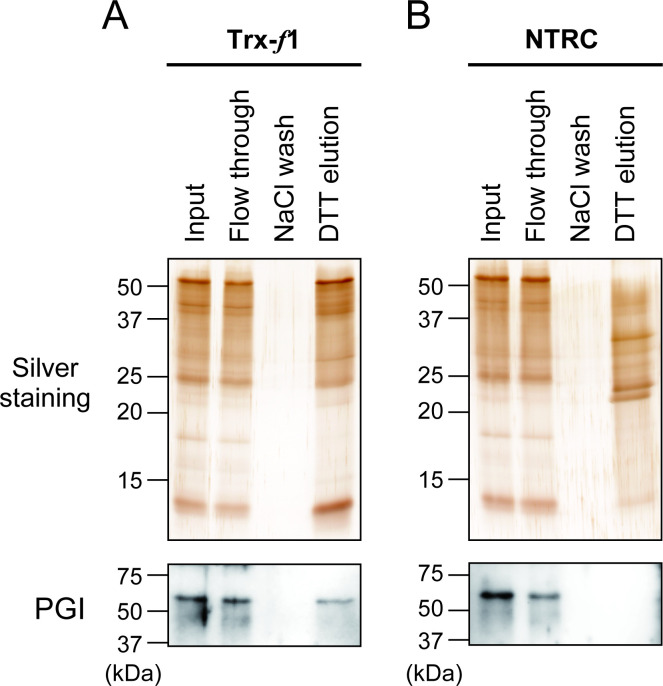
Identification of plastidial PGI as a Trx-interacting protein. *Arabidopsis* Trx-*f*1 (**A**) or NTRC (**B**) targets were screened using affinity chromatography from spinach chloroplasts. Eluted proteins were analyzed *via* SDS-PAGE with silver staining. PGI binding to Trx-*f*1 or NTRC was validated through immunoblotting analysis using an *Arabidopsis* PGI1 antibody. The uncropped and unedited immunoblotting images are shown in Supplementary Figure S1.

First, we investigated whether plastidial PGI physically interacts with Trx using an affinity chromatography-based screening method [14]. One plastidial Trx isoform from *Arabidopsis*, Trx-*f*1, was used as bait, with its active site sequence WCGPC mutated to WCGPS to stably capture target enzymes *via* intermolecular disulfide bonds. For comparison, a similar variant of NTRC was also used [22]. Soluble chloroplast proteins extracted from spinach leaves were loaded onto affinity columns immobilized with Trx-*f*1 or NTRC. Proteins bound to Trx-*f*1 or NTRC *via* intermolecular disulfide bonds were eluted using the reducing agent dithiothreitol (DTT). As previously demonstrated [22], the SDS-PAGE profiles of DTT-eluted proteins differed between experiments using Trx-*f*1 (Figure 1A) and NTRC (Figure 1B), indicating that Trx-*f* and NTRC employ different mechanisms for target recognition. Immunoblotting analyses revealed that PGI associates with Trx-*f*1, but not with NTRC (Figure 1, Supplementary Figure S1). These results suggest that plastidial PGI may be redox-regulated in a Trx-dependent manner.

### Plastidial PGI is reduced by specific Trx subtypes

**Figure 2:**
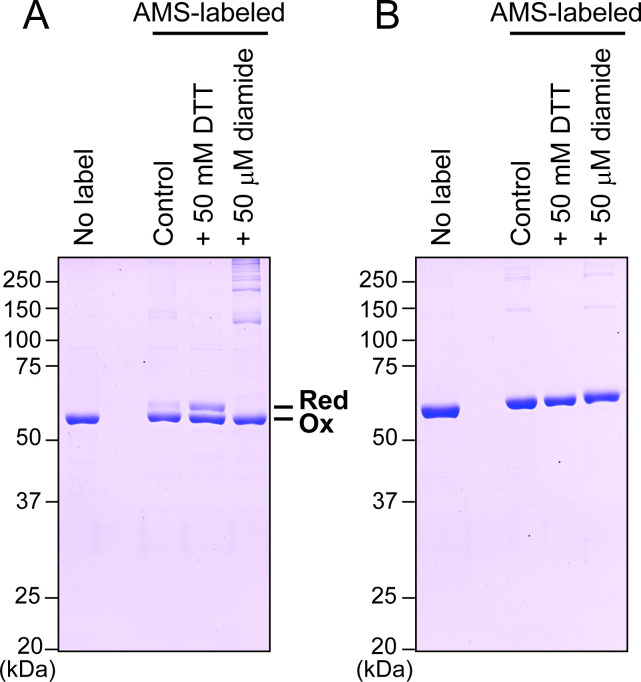
Redox sensitivity of purified PGI. PGI1 (**A**) and cytPGI (**B**) are shown. Each PGI (2 μM) was treated with 50 mM DTT (reductant) or 50 μM diamide (oxidant) for 30 min, followed by AMS labeling and analysis on nonreducing SDS-PAGE. Ox, oxidized form; Red, reduced form.

In *Arabidopsis*, two nuclear genes encode PGI isoforms: *At4g24620* for plastid-targeted PGI (PGI1) and *At5g42740* for cytosolic PGI (cytPGI). Their subcellular localizations were experimentally confirmed using fluorescent reporter-tagged proteins [19]. We prepared each PGI isoform as a recombinant protein without any affinity tag (Figure 2; see the ‘No label’ lanes). Initially, we examined the redox sensitivity of PGI1 and cytPGI using the thiol-modifying reagent, 4-acetamido-4′-maleimidylstilbene-2,2′-disulfonate (AMS). This method enabled the discrimination of redox state through protein mobility changes in non-reducing SDS-PAGE due to thiol modification [9]. PGI1 existed mainly in an oxidized state under control conditions but partially shifted to a reduced state in the presence of 50 mM DTT (Figure 2A). The band corresponding to the reduced state completely disappeared in the presence of the oxidant, 50 µM diamide. In contrast, cytPGI showed no redox state change following either reducing or oxidizing treatment (Figure 2B). These results indicate that only the plastid-localized PGI1 is redox-sensitive.

**Figure 3:**
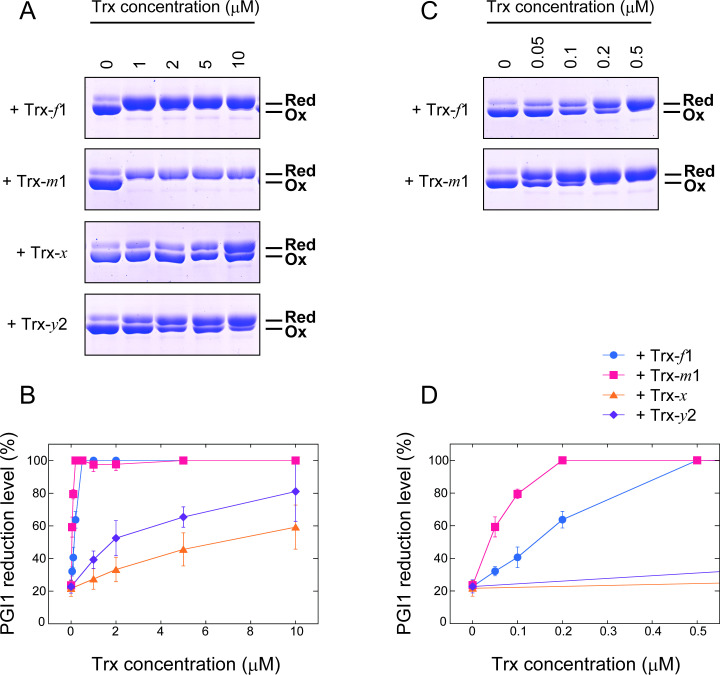
Trx subtype-dependent reduction of PGI1. PGI1 (2 µM) was incubated with various concentrations of Trx isoforms (A, B: 1–10 µM; C, D: 0.05–0.5 µM) in the presence of 0.2 mM DTT for 30 min. (**A, C**) PGI1 was labeled with AMS and loaded on nonreducing SDS-PAGE. Ox, oxidized form; Red, reduced form. (**B, D**) PGI1 reduction level was calculated as the ratio of the reduced state to the total. Each value represents the mean ± SD (3–9 technical replicates).

 Next, we examined Trx involvement in PGI1 reduction (Figure 3). PGI1 was incubated with varying concentrations (1–10 µM) of *Arabidopsis* Trx isoforms (Trx-*f*1, Trx-*m*1, Trx-*x,* or Trx-*y*2) in the presence of 0.2 mM DTT (Figure 3A and B). PGI1 was efficiently reduced by Trx-*f*1 or Trx-*m*1; only 1 µM of either isoform was sufficient to complete reduction. Further experiments using lower concentrations (0.05–0.5 µM) indicated that Trx-*m*1 was more effective in reducing PGI1 (Figure 3C and D). These results suggest that PGI1 redox modification strongly depends on specific Trx subtypes.

### Cys548 and Cys606 are essential for PGI1 redox modification

**Figure 4:**
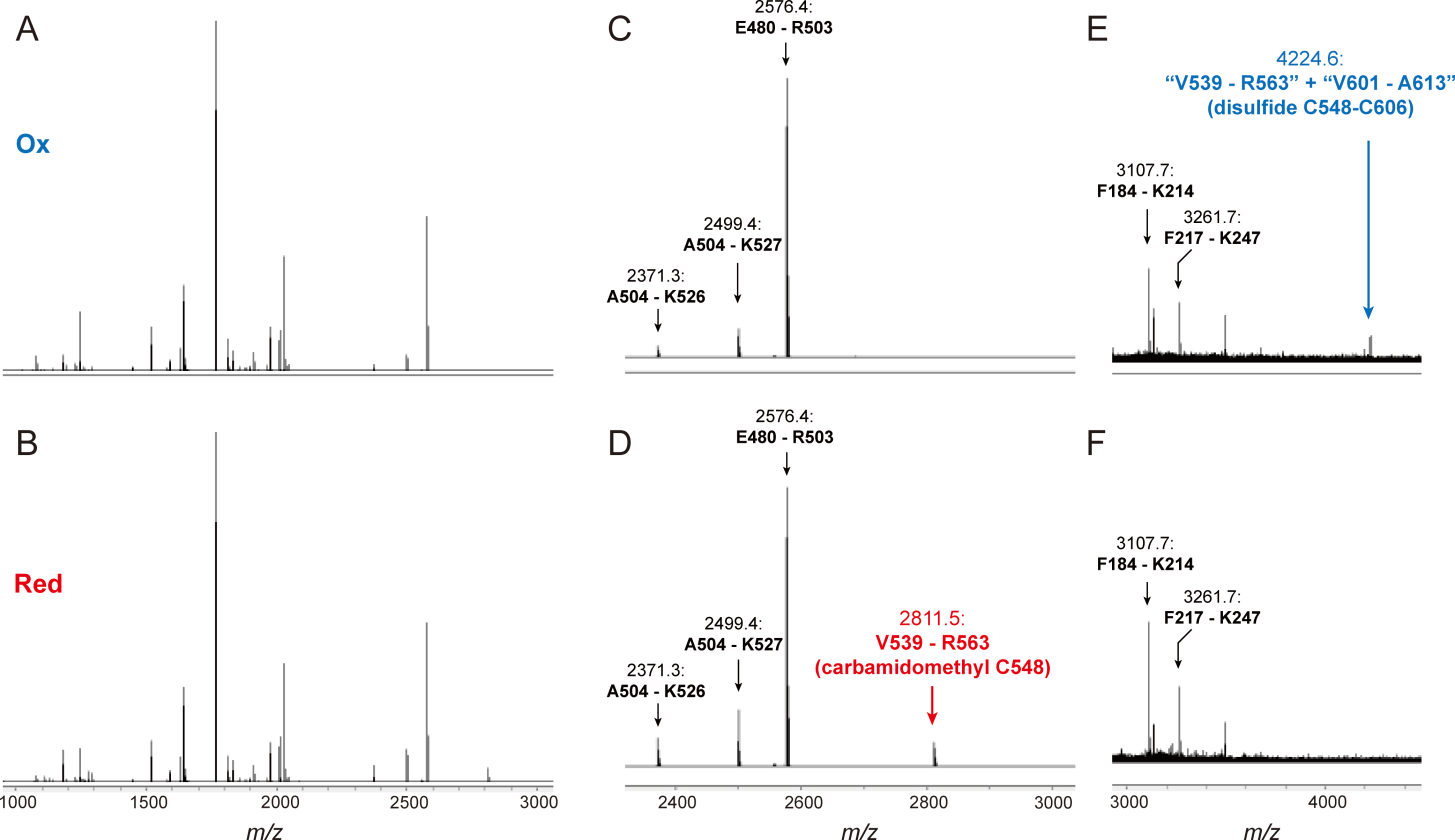
Identification of redox-active Cys residues in PGI1 *via* MS-based peptide mapping. Mass spectra of tryptic peptides from oxidized (Ox; **A, C, E**) and reduced (Red; **B, D, F**) PGI1 are shown. (**A, B**) Overall mass spectra. (C–F) Mass peaks specific to oxidized and reduced samples, highlighted in blue and red, respectively.

To identify Cys residues responsible for PGI1 redox modification, we performed MS-based peptide mapping (Figure 4). The oxidized PGI1 fraction was excised from an SDS-PAGE gel and incubated without (oxidized sample) or with (reduced sample) DTT, followed by Cys alkylation with iodoacetamide and in-gel digestion with trypsin. The mass spectra of the resulting peptides were compared between the oxidized and reduced samples. Overall, the spectra were largely similar (Figure 4A and B), although a few peaks were unique to each sample. A mass peak of 2811.5 appeared only in the reduced sample (Figure 4C and D). This peak corresponded to a Val539–Arg563 peptide containing carbamidomethylated Cys548. Conversely, a mass peak of 4224.6 was unique to the oxidized sample (Figure 4E and F). This peak matched the combined mass of two peptides: Val539–Arg563 and Val601–Ala613. As these peptides contain Cys548 and Cys606, respectively, the data suggest that these two Cys residues form a disulfide bond.

**Figure 5:**
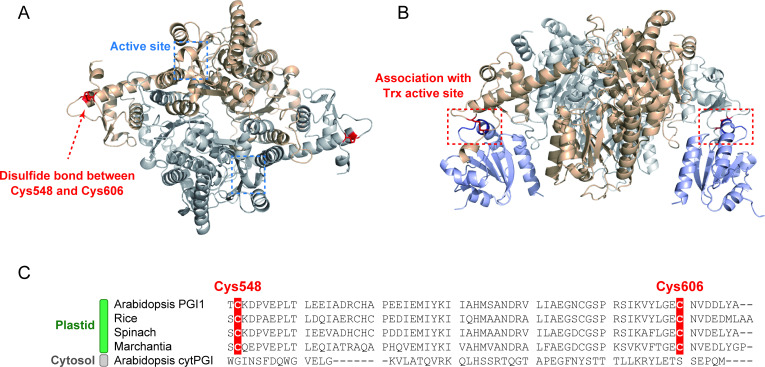
*In silico* structure and sequence analysis of PGI1. (**A**) AlphaFold3-predicted structure of PGI1 homodimer showing the Cys548–Cys606 disulfide bond. The location of the active site (constituting Gly194, Ser195, Ser246, Lys247, Ser248, Thr251, and Gln388 from one subunit and His421 from the other subunit) is shown based on previous structural studies [23,24]. (**B**) AlphaFold3-predicted protein complex of PGI1 homodimer with two Trx-*f*1 molecules (shown in light blue). Association between the disulfide bond in PGI1 and Trx-*f*1 active site (WCGPC motif) is highlighted. (**C**) Alignment of the C-terminal region of PGI proteins. Plastidial PGI (from *Arabidopsis*, rice, spinach, and *Marchantia*) and cytosolic PGI (from *Arabidopsis*) are shown. Alignment of full amino acid sequences is shown in Supplementary Figure S2.

 We then assessed the positions of Cys548 and Cys606 within PGI1. The functional unit of PGI is known to be a homodimer [23,24]. AlphaFold3-based prediction of the PGI1 homodimer three-dimensional structure indicated that intramolecular disulfide bonds between Cys548 and Cys606 form on the protein surface (Figure 5A). Structural modeling of a PGI1–Trx-*f* protein complex suggested that the disulfide bond between Cys548 and Cys606 is located near the Trx-*f* active site (Figure 5B). Sequence alignment showed that these Cys residues in *Arabidopsis* PGI1 are conserved in plastidial PGI from other terrestrial plants but not in redox-insensitive cytPGI (Figure 5C, Supplementary Figure S2).

**Figure 6:**
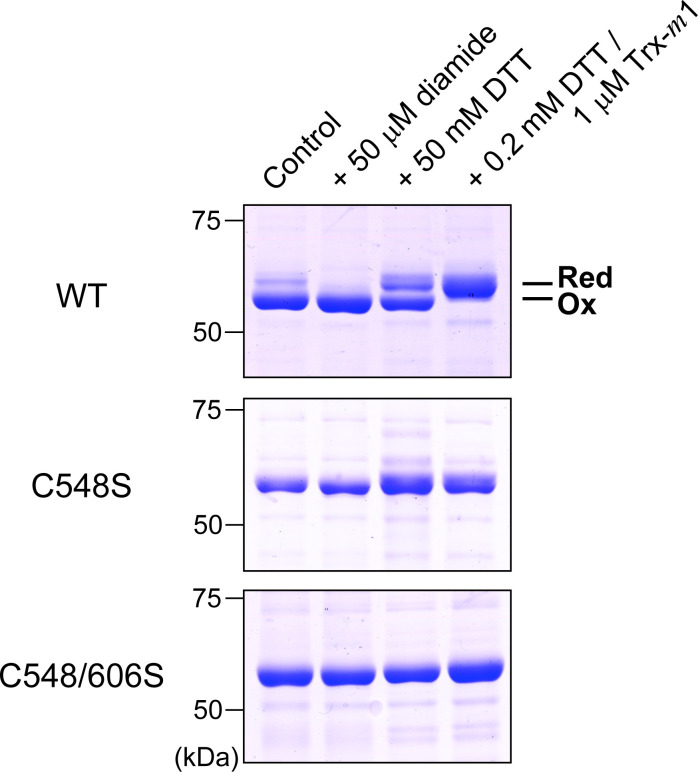
Involvement of Cys548 and Cys606 in PGI1 redox modification. Wildtype (WT) and Cys-substituted mutant (C548/606S) PGI1 (2 μM) were treated with the indicated oxidant or reductant (with Trx) for 30 min, followed by AMS labeling and analysis on nonreducing SDS-PAGE. Ox, oxidized form; Red, reduced form.

 We also performed site-directed mutagenesis, with Cys548 alone or both Cys548 and Cys606 in PGI1 replaced by Ser to create the C548S and C548/606S mutants, which were then tested for redox sensitivity (Figure 6). Although WT PGI1 showed clear redox shifts in response to reducing and oxidizing treatments, the C548S and C548/606S mutants lost such redox responsiveness. Collectively, these results demonstrate that Cys548 and Cys606 are essential for PGI1 redox modification.

### Light- and Trx-dependent PGI1 redox modification is evident *in vivo*

**Figure 7:**
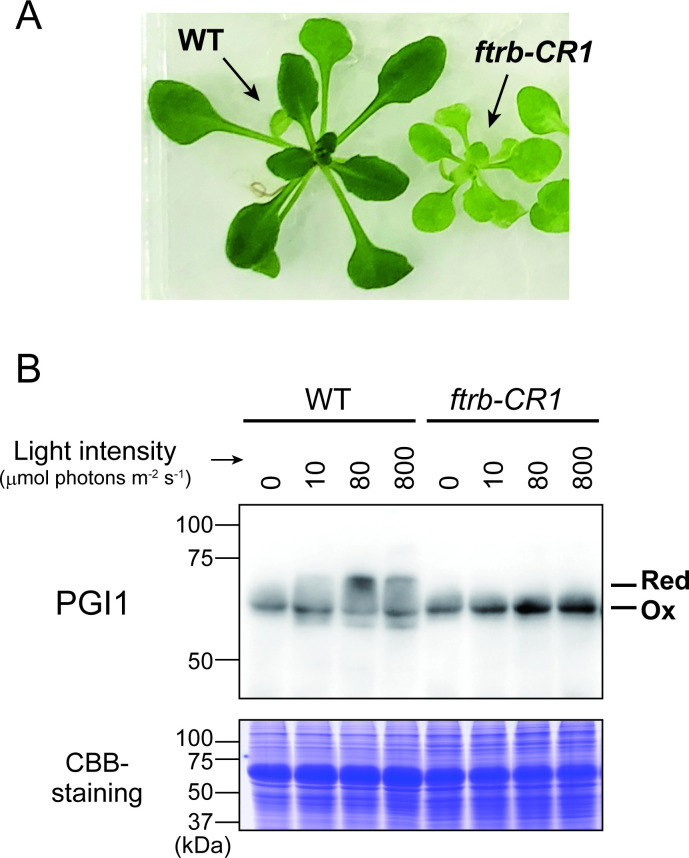
PGI1 redox state *in vivo*. (**A**) Growth phenotypes of *Arabidopsis* plants used in this experiment. WT, wildtype; *ftrb-CR1*, CRISPR/Cas9-based knockout mutant of FTR. (**B**) Plants were irradiated at the indicated light intensities for 20 min. Equal amounts of total leaf protein were loaded into each lane. As a loading control, the Rubisco large subunit was stained with Coomassie brilliant blue R-250 (CBB). Ox, oxidized form; Red, reduced form. The uncropped and unedited immunoblotting images are shown in Supplementary Figure S3.

We investigated whether PGI1 undergoes redox state changes in response to light *in vivo* using *Arabidopsis* plants. To assess the involvement of the Trx system, we also analyzed a mutant defective in the Fd/Trx pathway (Figure 7A). The *ftrb-CR1* mutant was generated using CRISPR/Cas9 to knock out FTR, a key component of the Fd/Trx pathway [25]. Redox shift assays using a PGI1-specific antibody showed that PGI1 was partially converted from the oxidized to reduced state upon light exposure in WT plants. In contrast, PGI1 remained oxidized regardless of light conditions in the *ftrb-CR1* mutant (Figure 7B, Supplementary Figure S3). These results suggest that PGI1 undergoes light-induced redox modification *in vivo*, dependent on the Fd/Trx pathway.

### PGI1 catalytic activity is insensitive to redox modification

**Figure 8:**
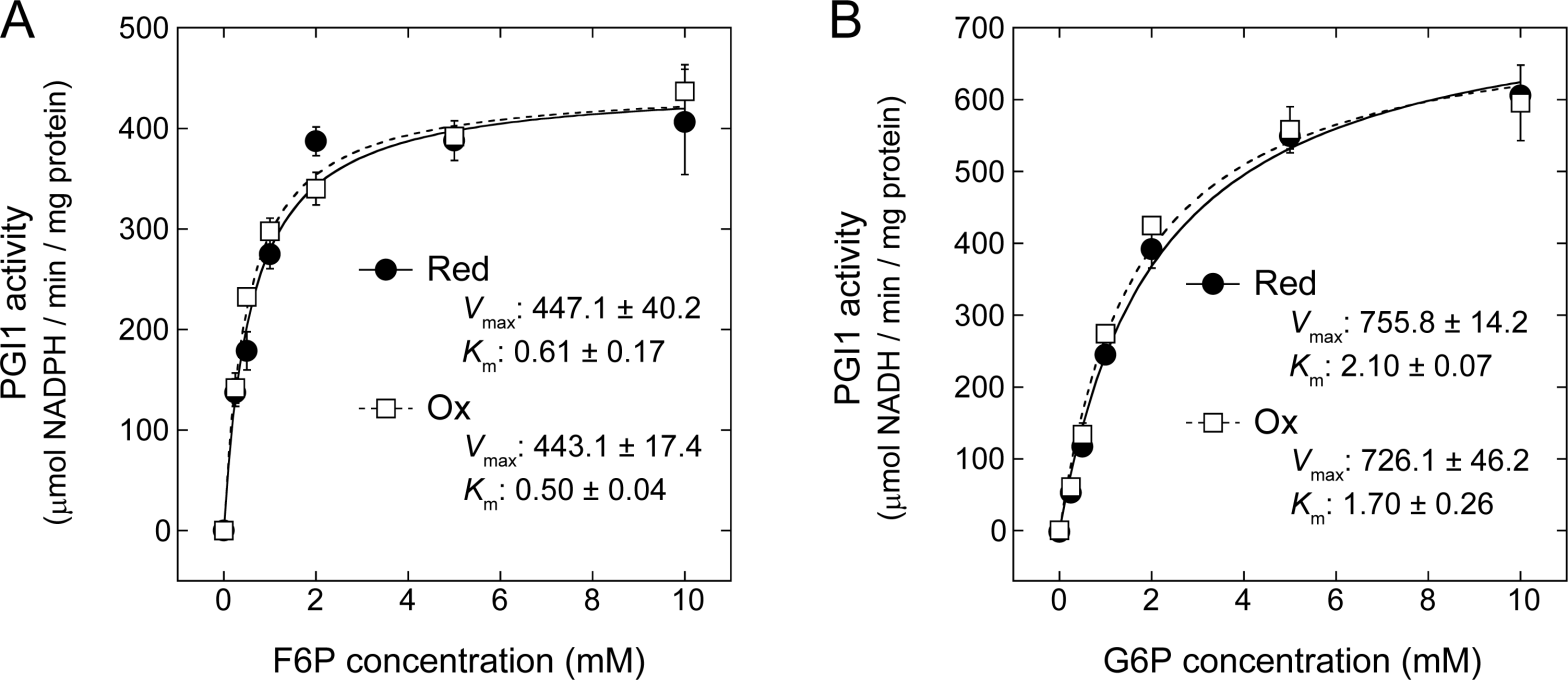
Michaelis–Menten curves for reduced (Red) and oxidized (Ox) states of PGI1. F6P (**A**) or G6P (**B**) was used as a substrate. Reduced state was prepared by incubating PGI1 (2 µM) with 1 µM Trx-*m*1 and 0.2 mM DTT; oxidized state was prepared by incubating PGI1 (2 µM) with 50 µM diamide. The kinetic parameters *V*_max_ and *K*_m_ are shown. Each value represents the mean ± SD (3 technical replicates).

We investigated whether redox modification affects PGI1 catalytic activity using recombinant PGI1 proteins. Figure 8 shows Michaelis–Menten plots using F6P (Figure 8A) or G6P (Figure 8B) as substrates. In both cases, PGI1 activity was unchanged between the reduced and oxidized forms. Correspondingly, the calculated *V*_max_ and *K*_m_ values were not largely different between the two redox states.

**Figure 9:**
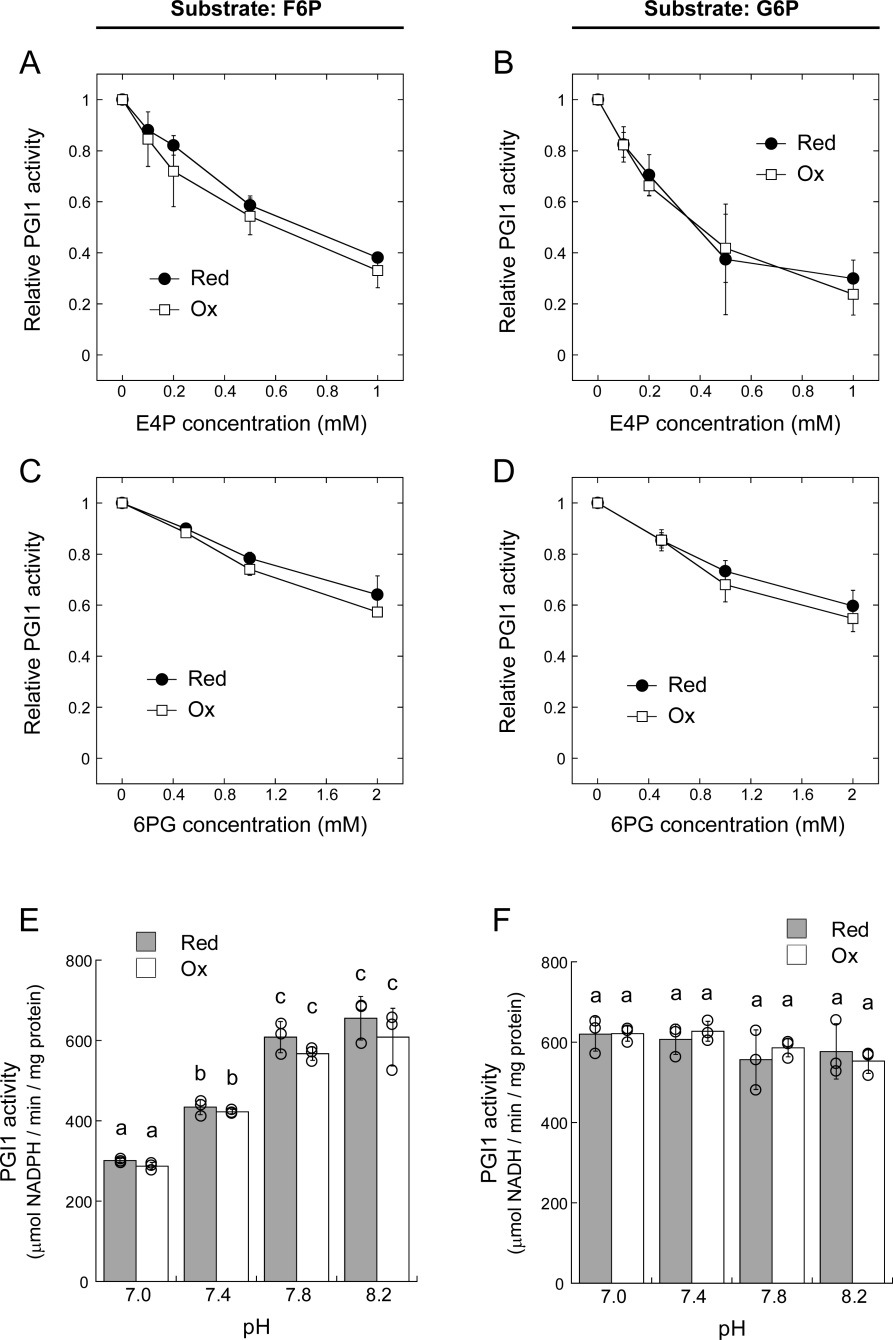
Effects of reduced (Red) and oxidized (Ox) states on metabolite and pH sensitivity of PGI1 catalytic activity. E4P (**A, B**), 6PG (**C, D**), and pH (**E, F**) effects are shown. F6P (**A, C, E**) or G6P (**B, D, F**) was used as a substrate. Reduced state was prepared by incubating PGI1 (2 µM) with 1 µM Trx-*m*1 and 0.2 mM DTT; oxidized state of PGI1 was prepared by incubating PGI1 (2 µM) with 50 µM diamide. Each value represents the mean ± SD (3–8 technical replicates). (**E, F**) Different letters denote significant differences (*P*<0.05, Tukey–Kramer multiple comparison test).

 PGI1 activity is known to be inhibited by certain metabolites, including erythrose 4-phosphate (E4P) and 6-phosphogluconate (6PG) [26,27]. Therefore, we tested whether redox states affect PGI1 sensitivity to these metabolites. Consistent with previous reports [26,27], PGI1 activity decreased with increasing concentrations of E4P (Figure 9A and B) and 6PG (Figure 9C and D). However, inhibition was indistinguishable between reduced and oxidized forms. We also examined the effect of pH on PGI1 activity and its redox sensitivity. When F6P was used as a substrate, PGI1 activity increased with rising pH (Figure 9E). In contrast, PGI1 activity remained constant across pH levels when G6P was used (Figure 9F). In both cases, the redox state did not affect the pH sensitivity of catalytic activity. Taken together, these findings indicate that PGI1 activity is unaffected by redox modification.

## Discussion

Prior redox-proteomics studies identified PGI1 as one of the enzymes that undergo redox state changes in response to light [16,17]. For example, Huang et al. [16] quantitatively assessed proteome-wide redox changes following excessive light exposure by differentially labeling reduced and oxidized Cys residues using light and heavy iodoacetamide. Additionally, an earlier proteomics study using a fluorescent thiol probe identified PGI1 as a Trx-interacting enzyme [28]. These findings suggest that PGI1 is subject to light-responsive, Trx-dependent redox regulation. However, such studies do not assess functional changes at the individual enzyme level; therefore, the redox regulation of PGI1 has remained inconclusive.

The present study demonstrates that PGI1, but not cytPGI, undergoes Trx-mediated redox modification both *in vitro* (Figures 2 and 3) and *in vivo* (Figure 7). We also showed that Trx-*m* and, to a lesser extent, Trx-*f* are effective in reducing PGI1 (Figure 3). This trend in Trx selectivity is commonly observed in many redox-regulated enzymes involved in plastid carbon metabolism [13]. It should be noted that other regulatory factors (e.g., glutaredoxin) may also be involved in PGI1 redox modification. However, activity assays yielded unexpected results: PGI1 catalytic activity was entirely unaffected by redox states, at least directly (Figure 8).

 The mechanisms underlying PGI1 regulation have been partially characterized. For example, the *K*_m_ for G6P is several times higher than that for F6P [27], which aligns with our data: *K*_m_ for F6P was approx. 0.5 mM, whereas *K*_m_ for G6P was approx. 2.0 mM (Figure 8). Additionally, we found that PGI1 activity converting F6P to G6P increases under alkaline pH, whereas the reverse reaction remains unchanged (Figure 9E and F). These PGI1 properties may play a role in directing carbon flow toward starch synthesis and limiting G6P backflow into the Calvin–Benson cycle, especially under photosynthetic conditions. We also confirmed the metabolic control of PGI1 activity (e.g., E4P inhibition; Figure 9A–D), as reported in previous studies [26,27]. Importantly, PGI1 redox state does not influence its sensitivity to either pH or metabolite inhibition (Figure 9).

 MS-based peptide mapping and site-directed mutagenesis analyses revealed that Cys548 and Cys606 form an intramolecular disulfide bond in PGI1 (Figures 4 and 6). AlphaFold3-based structural predictions suggest that these residues are exposed to the protein surface, enabling Trx accessibility (Figure 5). Previously, PGI active site was mapped using the X-ray crystal structure of wheat cytPGI bound to G6P [23,24]. In *Arabidopsis* PGI1, the active site appears to be spatially distant from the redox-active Cys pair (Figure 5A), making it unlikely that redox switching induces conformational changes at the active site or alters catalytic efficiency. Future research should focus on elucidating the structural basis for the redox insensitivity of PGI1 activity, by resolving the structure of PGI1 in each redox state at high resolution and directly comparing them.

 In plastid enzymes that are established targets of redox regulation (e.g., certain Calvin–Benson cycle enzymes and ATP synthase), changes in redox state are closely coupled with changes in enzymatic activity [29–32]. Most are activated through reductive disulfide bond cleavage. Exceptionally, G6P dehydrogenase [33,34] and phosphofructokinase [35] are activated by oxidative disulfide bond formation. To our knowledge, no plastid enzymes have been reported in which redox state changes occur without affecting catalytic activity. This could lead to the mistaken assumption that all enzymes identified by redox-proteomics undergo redox-dependent activity changes. Our study identifies PGI1 as the first plastid enzyme that undergoes Trx-mediated redox modification without any change in catalytic activity. The physiological significance of PGI1 redox modification remains to be elucidated; several possibilities (e.g., protein stability) should be tested in future. Nevertheless, this study underscores the importance of individual validation to achieve a robust understanding of the redox regulation system.

## Methods

### Preparation of expression plasmids

Total RNA was extracted from *Arabidopsis* as described previously [36] and used as a template for RT-PCR. Gene fragments encoding the predicted mature protein regions of PGI1 (At4g24620; Asp70–Ala613 [23]) or cytPGI (At5g42740; Met1–Met560) were amplified and inserted into the pET-23c expression vector (Novagen). PGI expression plasmid sequences were confirmed through DNA sequencing. Expression plasmids for *Arabidopsis* Trx isoforms were prepared previously [9,37].

### Site-directed mutagenesis

Point mutations in PGI1 (Cys548 to Ser and Cys606 to Ser) were introduced using the PrimeSTAR Mutagenesis Basal Kit (Takara) following the manufacturer’s instructions. The oligonucleotide primers used were as follows (mutated codons are underlined): for Cys548 to Ser, 5′-GCCACTAGTAAAGATCCGGTAGAGCCA-3′ (forward) and 5′-ATCTTTACTAGTGGCTTCATTAAGAAC-3′ (reverse); for Cys606 to Ser, 5′-GGCGAGAGCAATGTGGATGACCTGTAC-3′ (forward) and 5′-CACATTGCTCTCGCCCAAATACACTTT-3′ (reverse).

### Protein expression and purification

Each PGI plasmid was transformed into *Escherichia coli* Rosetta (DE3) pLysS cells. Cultures were grown at 37°C, and protein expression was induced by adding 0.5 mM isopropyl-1-thio-β-_D_-galactopyranoside, followed by incubation overnight at 21°C. Collected cells were used for protein purification.

 To purify proteins, *E. coli* cells were suspended in 25 mM Tris-HCl (pH 7.5), 1 mM EDTA, and 0.5 mM DTT and subsequently disrupted *via* sonication. Lysates were centrifuged at 125,000×g for 40 min. Supernatants were then applied to a DEAE-Toyopearl 650 M column (Tosoh), and proteins were eluted using a 0–200 mM linear NaCl gradient in the abovementioned buffer. PGI-containing fractions were verified through SDS-PAGE. If necessary, protein samples were further purified on a Butyl-Toyopearl 650 M column (Tosoh). Proteins were eluted with a 30–0% (percent saturation) inverse gradient of ammonium sulfate in the abovementioned buffer. Purified PGI proteins were dialyzed in 25 mM Tris-HCl (pH 7.5) and concentrated using Amicon Ultra filters (50 kDa cutoff; Merck). Protein concentration was determined using the Bradford assay (Bio-Rad) with bovine serum albumin employed as a standard. Purified PGI1 was also used as an antigen to generate an antibody.

### Screening for Trx-*f*1- or NTRC-interacting proteins

Trx affinity chromatography [14] was used to identify Trx-*f*1- or NTRC-interacting proteins from spinach chloroplasts. *Arabidopsis* Trx-*f*1 and NTRC were expressed as monocysteine variants. NTRC has two redox-active Cys pairs (one each in the NTR and Trx domains); the pair in the Trx domain was mutated to a single Cys. Detailed methods are provided in Refs 22,38. PGI binding was verified *via* immunoblotting using the *Arabidopsis* PGI1 antibody.

### Redox shift assay of PGI *in vitro*

For reduction or oxidation, PGI was incubated at room temperature in 50 mM Tris-HCl (pH 7.5) and 50 mM NaCl. Protein concentrations and reaction durations are shown in the figure legends. Proteins were precipitated with 10% (w/v) trichloroacetic acid and washed with ice-cold acetone. Thiol groups were labeled with AMS (Invitrogen) for 1 h at room temperature. Samples were analyzed *via* non-reducing SDS-PAGE and stained with Coomassie Brilliant Blue R-250 (CBB). PGI redox states were identified through mobility shifts due to thiol modification.

### MS-based peptide mapping analysis

After AMS labeling and nonreducing SDS-PAGE, CBB-stained oxidized PGI1 bands were excised and destained in 50 mM NH_4_HCO_3_ and 50% (v/v) acetonitrile. For reduction, gel slices were incubated in 100 mM NH_4_HCO_3_ with 10 mM DTT at 56°C for 1 h. Free thiols were alkylated with iodoacetamide. After drying, gel slices were incubated in 50 mM NH_4_HCO_3_ containing 20 ng/μl trypsin (Promega) at 37°C overnight. Peptides were extracted with 0.1% (v/v) trifluoroacetic acid and 50%–75% (v/v) acetonitrile, and then mixed with α-cyano-4-hydroxycinnamic acid on a MALDI plate (MTP 384 target plate ground steel BC, Bruker Daltonics). Mass spectra were obtained using MALDI-TOF MS (UltrafleXtreme; Bruker Daltonics). The measurement mode was set to positive reflector mode. Calibration was performed using Peptide Calibration Standard II (Bruker Daltonics). Results were queried with the Mascot search engine from Matrix Science to identify matched peptides. Following parameters were used for Mascot database searches: database; Araport11, enzyme; trypsin (up to 1 missed cleavages), fixed modifications; carbamidomethyl (C), variable modifications; oxidation (M), mass values; monoisotopic, peptide mass tolerance; 50 ppm.

### Structure modeling of PGI1

Three-dimensional structure of PGI1 was predicted using AlphaFold3. The enzyme was modeled as a homodimer, which was reported to be its functional unit [23,24]. A complex model including the PGI1 homodimer and two Trx-*f*1 molecules was also predicted.

### PGI1 catalytic activity measurements

PGI1 catalytic activity was assayed at 25°C using coupling reactions and spectrophotometry.

For the F6P to G6P conversion reaction, PGI1 activity was measured in solution containing 50 mM Tris-HCl (pH 7.5), 50 mM NaCl, 5 mM MgCl_2_, 1 mM NADP^+^, 8 U G6P dehydrogenase (from *Leuconostoc mesenteroides*; Sigma-Aldrich), varying F6P concentrations (described in each figure), and 660 ng of PGI1. Absorbance at 340 nm was monitored to measure NADP^+^ reduction. A molar extinction coefficient of 6.2 mM^−1^ cm^−1^ for NADPH was used.

For the G6P to F6P conversion reaction, PGI1 activity was measured in solution containing 50 mM Tris-HCl (pH 7.5), 50 mM NaCl, 5 mM MgCl_2_, 0.5 mM ATP, 0.2 mM NADH, 1 U F6P kinase (from *Bacillus stearothermophilus*; Sigma-Aldrich), 1–2 U aldolase (from rabbit muscle; Sigma-Aldrich), 50–130 U triosephosphate isomerase (from rabbit muscle; Sigma-Aldrich), 5–13 U glycerol 3-phosphate dehydrogenase (from rabbit muscle; Sigma-Aldrich), varying G6P concentrations (described in each figure), and 660 ng of PGI1. Absorbance at 340 nm was monitored to measure NADH oxidation. A molar extinction coefficient of 6.2 mM^−1^ cm^−1^ for NADH was used.

The kinetic parameters *K*_m_ and *V*_max_ were obtained by fitting data to the Michaelis–Menten equation using KaleidaGraph (Hulinks Inc.). To test metabolite sensitivity, varying concentrations of E4P or 6PG were added. For pH sensitivity, the reaction buffer was adjusted to pH 7.0, 7.4, 7.8, or 8.2 using 50 mM Tris-HCl. These pH conditions were confirmed not to affect the activity of enzymes used in the coupling assays.

### Determination of PGI1 redox state *in vivo*

*Arabidopsis* Col-0 (WT) and *ftrb-CR1* mutant [25] were grown on half-strength Murashige and Skoog medium with 2% (w/v) sucrose in a growth chamber (80 μmol photons m^−2^ s^−1^, 22°C, and 16/8 h light/dark) for three weeks. After 20 min exposure to the indicated light intensity, PGI1 redox states were determined as described previously [39] using the *Arabidopsis* PGI1 antibody.

### Statistical analysis

Statistical analyses were performed with SPSS 12.0J software (SPSS Inc.) for the Tukey-Kramer multiple comparison test.

## Supplementary material

online supplementary material 1.

## Data Availability

All data are contained within the article.

## Open Access

This article has been published open access under our Subscribe to Open programme, made possible through the support of our subscribing institutions, learn more here: https://portlandpress.com/pages/open_access_options_and_prices#conditional

## Abbreviations

AMS: 4-acetamido-4’-maleimidylstilbene-2,2’-disulfonate
CBB: Coomassie Brilliant Blue R-250
DTT: dithiothreitol
E4P: erythrose 4-phosphate
F6P: fructose 6-phosphate
FTR: ferredoxin-thioredoxin reductase
Fd: ferredoxin
G6P: glucose 6-phosphate
NTRC: NADPH-thioredoxin reductase C
6PG: 6-phosphogluconate
PGI: phosphoglucose isomerase
Trx: thioredoxin
