# Supplementary material for: Plastidial phosphoglucose isomerase undergoes thioredoxin-mediated redox modification without altering catalytic activity
Source: Biochem J. 2025 Dec 17;482(24):1845–57. doi: 10.1042/BCJ20253425 (PMC12751066; doi:10.1042/BCJ20253425)
Supplement: online supplementary material 1. [file bcj-482-24-BCJ20253425-s001.pdf]

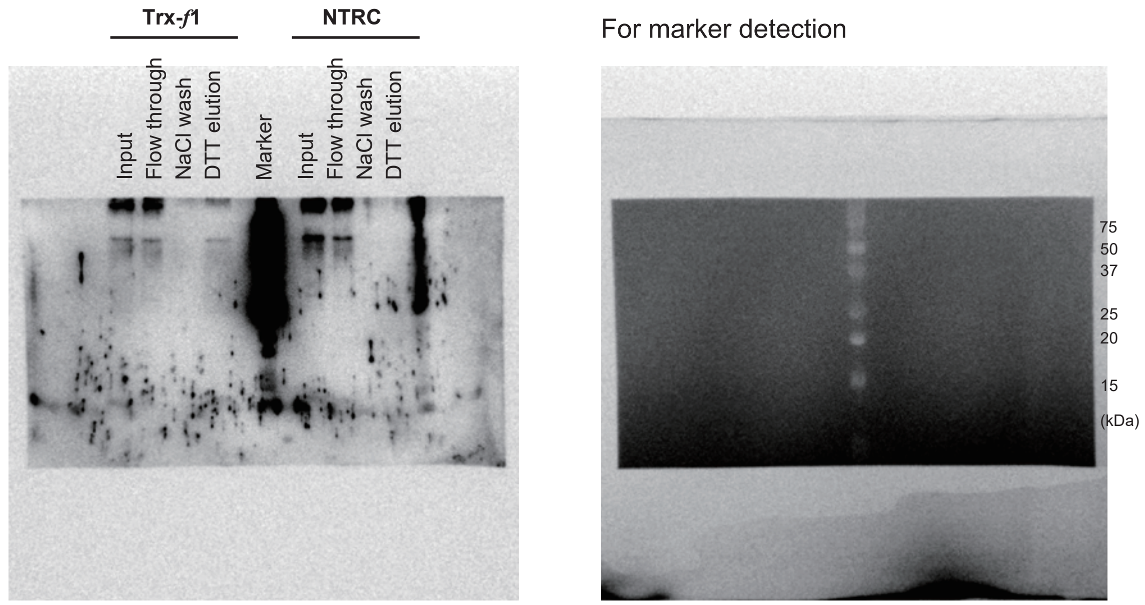

Supplementary Figure S1. Uncroped and unedited immunoblotting image for Figure 1.

```
Arabidopsis PGI1      ---MAS---L SGLYSSSPSL KPAKNHS--F KALPAQSRD- SFSFPHTS-- ---KPTNLPL T-LSSARSVA RDISHAD--- ---------- -SKKELLKDP
Rice                  ---MASISGA AAPPSSSAAC RLRLRRQLLL RPSHLRLRA- PHSIADLSRS SSSSSEQQPS S-TPAAPLAS RPGQNGS--- ---------- -TRGAVEKDP
Spinach               ---MASS--L SNLYSST-SL KPKISHLPTI TKNPISGPK- SLSFKPISSV ARDTPADLST S-SSSTNNL  PSLQKKK--- ---------- -ADGSLEKDP
Marchantia            MACISSRLSV SPNFSGLRKA LPSTSTSSTT VSVSSSGSEG SYAAGRLSLQ VVASAKRFPA SSTPSSPSQT PPVNAISSKV PEELVSTSAL PTKPVLETDP
Arabidopsis cytPGI    ---MASS--- TALICDTEAW KDLKGHVEDI K--------- ---------- ---------- ---------- ---------- ---------- ---KTHLRDL

                                                                                                                         *
Arabidopsis PGI1      DALWKRYLDW FYQQKELGLY LDISRVGFTD EFVAEMEPRF QAAFKAMEDL EKGSIANPDE GRMVGHYWLR NSK----LAP KPTLKTLIEN TLDSICAFSD
Rice                  IKLWERYVEW LYQHKELGLF VDVSRMGFTE EFLRRMEPRM QRAFAAMREL EKGAIANPDE GRMVGHYWLR DPG----LAP NSFLRTKIET TLDRILAFSQ
Spinach               RALWARYVEW LYQHKDLGLY LDVSRIGFSD EFVKEMEPRF EKAFKHMEGI EKGAIANPDE GRMVGHYWLR NSS----LAP TTFLKNQIDV TLDRVWQFAN
Marchantia            LKLWQRYLEW LYQDKDIGIE IDVSRIGFTD EYLAMMRPKL DQAFRAMADL EGGSIANPDE GRMVGHYWLR KPE----LAP TAYLQKQIVQ TIESVQSFAK
Arabidopsis cytPGI    MSDANRCQSM MMEFDGLLLD YSRQRATVET MDKLLNLAKA SQLTEKISRM FNGEHINSTE NRSVLHVALR APKDAVIKAD GMNVVPEVWN VLDKIKEFSD

Arabidopsis PGI1      DIISGKIKPP SSPEGRFTQI LSVGIGGSAL GPQFVAEALA PDNPPLK--- ---IRFIDNT DPAGIDHQIA QLGPELASTL VVVISKSGGT PETRNGLLEV
Rice                  DVVSGKIKPP SSPAGRFTQI LSIGIGGSAL GPQFVSEALA PDNPPLK--- ---IRFIDNT DPAGIDHQIA QLGPELASTL VIVISKSGGT PETRNGLLEV
Spinach               DVISGKIKAP TG--ERFTHI LSVGIGGSAL GPQFVAEALA PDNPPLK--- ---IRFIDNT DPAGIDHQIA QLGPELATTL VMVISKSGGT PETRNGLLEV
Marchantia            DVISGKIKPP PCPNGRFTQI LSVGIGGSAL GPQFVAEALA PDNPPLK--- ---LRFIDNT DPAGIDHQIA QLGPELSSTL VVVISKSGGT PETRNGLLEV
Arabidopsis cytPGI    KIRSGSWVGA TG--KPLKDV IAIGIGGSFL GPLFVHTALQ TDPEALESAK GRQLRFLANI DPVDVARNIS GLNPETTLVV VVSKTFTTAE TMLNARTLRE

                                                                                                                           *
Arabidopsis PGI1      QKAFREAGLN FAKQGVAITQ ENSLLDNTAR IEGWLARFPM YDWVGGRTSI MSAVGLLPAA LQG--INVRE MLTGAALMDE ATRTTSIKNN PAALLAMCWY
Rice                  QKAFRDAGLD FSKQGVAVTQ ENSLLDNTAR IEGWLARFPM FDWVGGRTSE MSAVGLLPAA LQG--IDIKE MLVGAALMDE ETRNTVVKEN PAALLALCWY
Spinach               QKAFRDAGLV FAKQGVAITQ ENSLLDNTAR IEGWIDRFPM FDWVGGRTSE MSAVGLLPAA LQG--IDIKE MLAGAALMDE ATKIPVLRSN PAALLAMSWY
Marchantia            QRAFREKGLD FSKQGVAITQ ENSLLDNTAR IEGWLARFPM YDWVGGRTSE MSAVGLLPAA LQG--IDIHG MLEGARLMDE TTRIRDLKTN PAALLALSWY
Arabidopsis cytPGI    WITAALGASA VAKHMVAVST NLALVEKFGI DP--NNAFAF WDWVGGRYSV CSAVGVLPLS LQYGFSMVEK FLKGASSIDQ HFQSTPFEKN IPVLLGLLSV

Arabidopsis PGI1      WASNGVGS-- ---------K DMVVLPYKDS LLLFSRYLQQ LVMESLGKEF DLDGN--TVN QGLTVYGNKG STDQHAYIQQ LRDGVHNFFA TFIEVLRDRP
Rice                  WASEGIGN-- ---------K DMVVLPYKDS LLLLSRYLQQ LVMESLGKEF DLDGN--RVN QGLTVYGNKG STDQHAYIQQ LREGVHNFFV TFIEVLRDRP
Spinach               WASDGVGS-- ---------K DMVVLPYKDS LLLFSRYLQQ LVMESLGKEF DLDGN--KVN QGLTVYGNKG STDQHAYIQQ LRDGVHNFFA TFIEVLRDRP
Marchantia            WATDGNGSKV GICSPHILVE DMVVLPYKDS LLLFSRYLQQ LVMESLGKEY DLDGN--LVN QGLAVYGNKG STDQHAYIQQ LREGVPNFFA TFIEVLRDRP
Arabidopsis cytPGI    WNVSFLGY-- ---------P ARAILPYSQA LEKFAPHIQQ VSMESNGKGV SIDGLPLPFE TGEIDFGEPG TNGQHSFYQL IHQGR-VIPC DFIGIVKSQQ

                         *
Arabidopsis PGI1      PGHDWELEPG VTCGDYLFGM LQGTRSALYA NGRESISVTI QEVTPTSVGA IIALYERAVG LYASIVNINA YHQPGVEAGK KAAAEVLALQ KRVLSVLNEA
Rice                  PGHDWELEPG VTCGDYLFGM LQGTRSALYS NDRESISVTV QEVTPRAVGA LVALYERAVG IYASLVNINA YHQPGVEAGK KAAGEVLALQ KRVLTVLNEA
Spinach               PGHDWELEPG VTCGDYLFGM LQGTRSALYA NNRESISVTV QEVTPRSVGA MVALYERAVG LYASLVNINA YHQPGVEAGK KAAAEVLALQ KRVLAVLNEA
Marchantia            PGHDWELEPG VTCGDYLFGM LQGTRQALYA NKRESITVTV NEVNPITVGA LIALYERAVG LYASLINVNA YHQPGVEAGK KAAGEVLALQ KRVLSVLNDA
Arabidopsis cytPGI    P-VYLKGEVV SNHDELMSNF FAQPDALAYG KTPEQLQK-- -ENVSENLIP HKTFSGNRPS LSLLLPELTA YN-------- --VGQLLAIY EHRVAVQGFV

                              *                                *
Arabidopsis PGI1      TCKDPVEPLT LEEIADRCHA PEEIEMIYKI IAHMSANDRV LIAEGNCGSP RSIKVYLGEC NVDDLYA--
Rice                  SCKDPAEPLT LDQIAERCHC PEDIEMIYKI IQHMAANDRA LIAEGSCGSP RSIKVYLGEC NVDEDMLAA
Spinach               SCKDPVEPLT IEEVADHCHC PDDIEMIYKI IAHMAANDRV ILAEGDCGSP RSIKAFLGEC NVDELYA--
Marchantia            SCQEPVEPLT LEQIATRAQA PHQVEMIYKI VAHMVANDRA LFAEGDCGSP KSVKVFTGEC NVEDLYGP-
Arabidopsis cytPGI    WGINSFDQWG VELG------ -KVLATQVRK QLHSSRTQGT APEGFNYSTT TLLKRYLETS SEPQM----
```

Supplementary Figure S2. Alignment of PGI amino acid sequences. Plastidial PGI (from *Arabidopsis*, rice, spinach, and *Marchantia*) and cytosolic PGI (from *Arabidopsis*) are shown. Two Cys residues involved in disulfide bond formation are highlighted by red. Other Cys residues conserved in Arabidopsis PGI1 are indicated by asterisks.

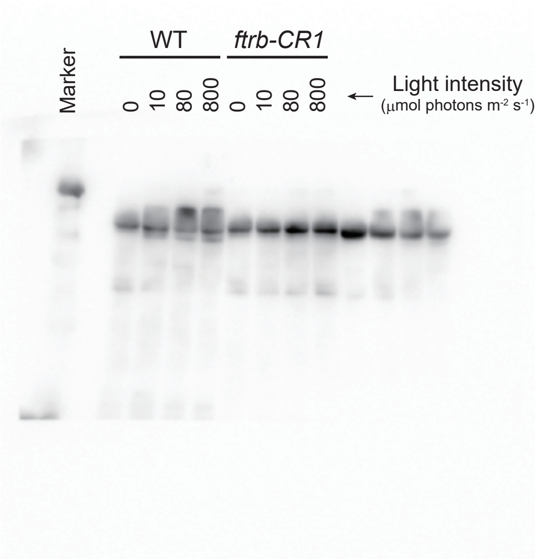

For marker detection

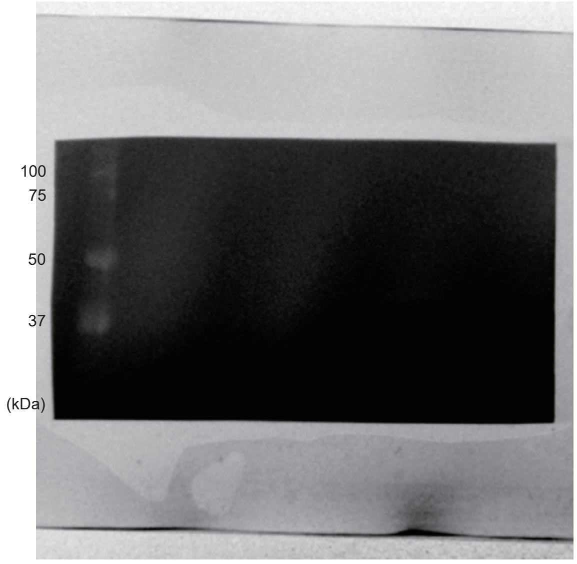

Supplementary Figure S3. Uncroped and unedited immunoblotting image for Figure 7B.
